# Founder mutation in the PMM2 promotor causes hyperinsulinemic hypoglycaemia/polycystic kidney disease (HIPKD)

**DOI:** 10.1002/mgg3.1674

**Published:** 2021-04-03

**Authors:** Sumaya Islam, Mehmet Tekman, Sarah E. Flanagan, Lisa Guay‐Woodford, Khalid Hussain, Sian Ellard, Robert Kleta, Detlef Bockenhauer, Horia Stanescu, Daniela Iancu

**Affiliations:** ^1^ Department Renal Medicine University College London London UK; ^2^ Institute of Biomedical and Clinical Science University of Exeter Medical School Exeter UK; ^3^ Center for Translational Research Children’s National Hospital Health System Washington DC USA; ^4^ Department of Endocrinology Sidra Medicine Doha Qatar; ^5^ Great Ormond Street Hospital for Children NHS Foundation Trust London UK

**Keywords:** founder effect, hyperinsulinism, hypoglycaemia, *PMM2* gene, polycystic kidney disease, promoter

## Abstract

**Background:**

Polycystic kidney disease with hyperinsulinaemic hypoglycaemia (HIPKD) is a recently described disease caused by a single nucleotide variant, c.‐167G>T, in the promoter region of *PMM2* (encoding phosphomannomutase 2), either in homozygosity or compound heterozygosity with a pathogenic coding variant in *trans*. All patients identified so far are of European descent, suggesting a possible founder effect.

**Methods:**

We generated high density genotyping data from 11 patients from seven unrelated families, and used this information to identify a common haplotype that included the promoter variant. We estimated the age of the promoter mutation with *DMLE*+ software, using demographic parameters corresponding to the European population.

**Results:**

All patients shared a 0.312 Mb haplotype which was absent in 503 European controls available in the 1000 Genomes Project. The age of this mutation was estimated as 105–110 generations, indicating its occurrence around 600 BC, a time of intense migration, which might explain the presence of the same mutations in Europeans around the globe.

**Conclusion:**

The shared unique haplotype among seemingly unrelated patients is consistent with a founder effect in Europeans.

## Graphical abstract

We identified a founder effect for the specific promoter variant that causes polycystic kidney disease and Hyperinsulinsim (HIPKD). The variant arose approximately 2500 years ago in Central Europe.

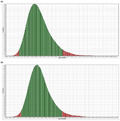

## 1 INTRODUCTION

In 2017, we described a previously unrecognised autosomal recessive disorder, polycystic kidney disease with hyperinsulinaemic hypoglycaemia (HIPKD) (Cabezas et al., 2017). All 17 patients from 11 unrelated families of European descent carry the same mutation, c.‐167G>T, on at least one *PMM2* allele (Cabezas et al., 2017). Since then, a few more cases have been described, all from a European background (Moreno Macián F et al., 2020). *PMM2* (OMIM #601785) encodes phosphomannomutase 2, a key enzyme involved in N‐glycosylation and bi‐allelic pathogenic coding variants in *PMM2* cause congenital disorder of glycosylation type 1a (CDG1a) (OMIM #212065), a multi‐system disorder which invariably includes neurological impairment and typically an abnormal transferrin isoelectric focusing test (Andreotti et al., 2015; Noreau et al., 2014; Sparks & Krasnewich, 2015; Westphal et al., 2001). In contrast, HIPKD primarily affects the kidneys and pancreatic beta cells with additional liver manifestations in the form of cysts or ductal plate malformation observed in some patients. Patients typically present with hypoglycaemia and multiple renal cysts in early childhood. The presence of a specific promoter variant, c.‐167G>T, on at least one allele of *PMM2* is the distinguishing genetic factor between the two diseases and is located in a region previously identified as a binding site for the transcription factor ZNF143 (Anno et al., 2011). We hypothesized that the mutation reduces the PMM2 expression in an organ‐specific manner as a result of impaired ZNF143 binding and altered functional genomic interactions with this site (Cabezas et al., 2017). Here we investigate the origin of the promoter variant and whether it relates to a common founder effect.

## 2 METHODS

### 2.1 Patients

We previously generated genotyping data obtained for 11 HIPKD patients from seven families, all part of the cohort presented by Cabezas et al., (2017). In addition, we included data from 18 parents, of which 12 are carrying the promoter variant c.‐167G>T. Of the 11 patients, four carried the promoter variant in a homozygous state, while the remaining seven were heterozygous, with a pathogenic coding variant in *PMM2 in trans*. The four homozygous HIPKD patients are offspring of a consanguineous family, resulting from first cousin marriages (Cabezas et al., 2017). All patients provided informed consent and the relevant ethics committees approved the genetic study.

All of the families in this study are of European descent: 3 families reside in Spain, 3 in the United Kingdom, and one living in the United States (Cabezas et al., 2017).

### 2.2 Whole genome SNP genotyping

The Spanish families had been genotyped on the *HumanOmni1S*‐*8v1_H* and *HumanOmni25M*‐*8v1*‐*1_B* chip, while the British/US families had been genotyped on the *HumanCyto*‐*12 chip*. Therefore, results were analysed separately according to the genotyping platform. Data were filtered for genotype errors and non‐informative markers as described previously (Abdelhadi et al., 2016; Bockenhauer et al., 2009; Cabezas et al., 2017).

### 2.3 Haplotype analysis

Based on genotyping data, haplotype reconstruction was performed as described previously (Rowczenio et al., 2017), separately for the Spanish and British/US sets. Detection of the common disease haplotype in the HIPKD patients and their parents was carried out separately for the two sets of data.

The genetic position of the markers was determined using The Human Genome Working Draft (https://genome.ucsc.edu/) and genetic distances calculated on the basis that 1 million base pairs is equivalent to 1 centiMorgan (Kent et al., 2002). To ease the analysis, the reference allele was labelled as (1) and the alternate allele as (2).

Control haplotypes were obtained from the latest release of the 1000 Genomes Project (The, 1000 Genomes Project Consortium, 2015). The allele frequencies and haplotypes of 503 European samples were used in this analysis.

### 2.4 Estimating the age of the promoter variant in HIPKD

Analysis was performed as described previously, using DMLE+version 2.3 software (www.dmle.org) (Abdelhadi et al., 2016; Reeve & Rannala, 2002).

The population parameters obtained were as follows: (1) the population growth rate was estimated to be 0.109 per generation using historical data for the population size of Europe in 1916 and 2016; (2) the proportion of the population sampled (*f*) was calculated as previously described (Rannala & Slatkin, 1998). This was estimated to be 1.76 × 10^−5^ for the Spanish set and 8.82 × 10^−6^ for the British/US set. The frequency of the disease allele in the European population was estimated to be about 1/1,000,000 based on the number of patients that have been diagnosed with HIPKD so far.

## 3 RESULTS

### 3.1 Haplotype analysis

**FIGURE 1:**
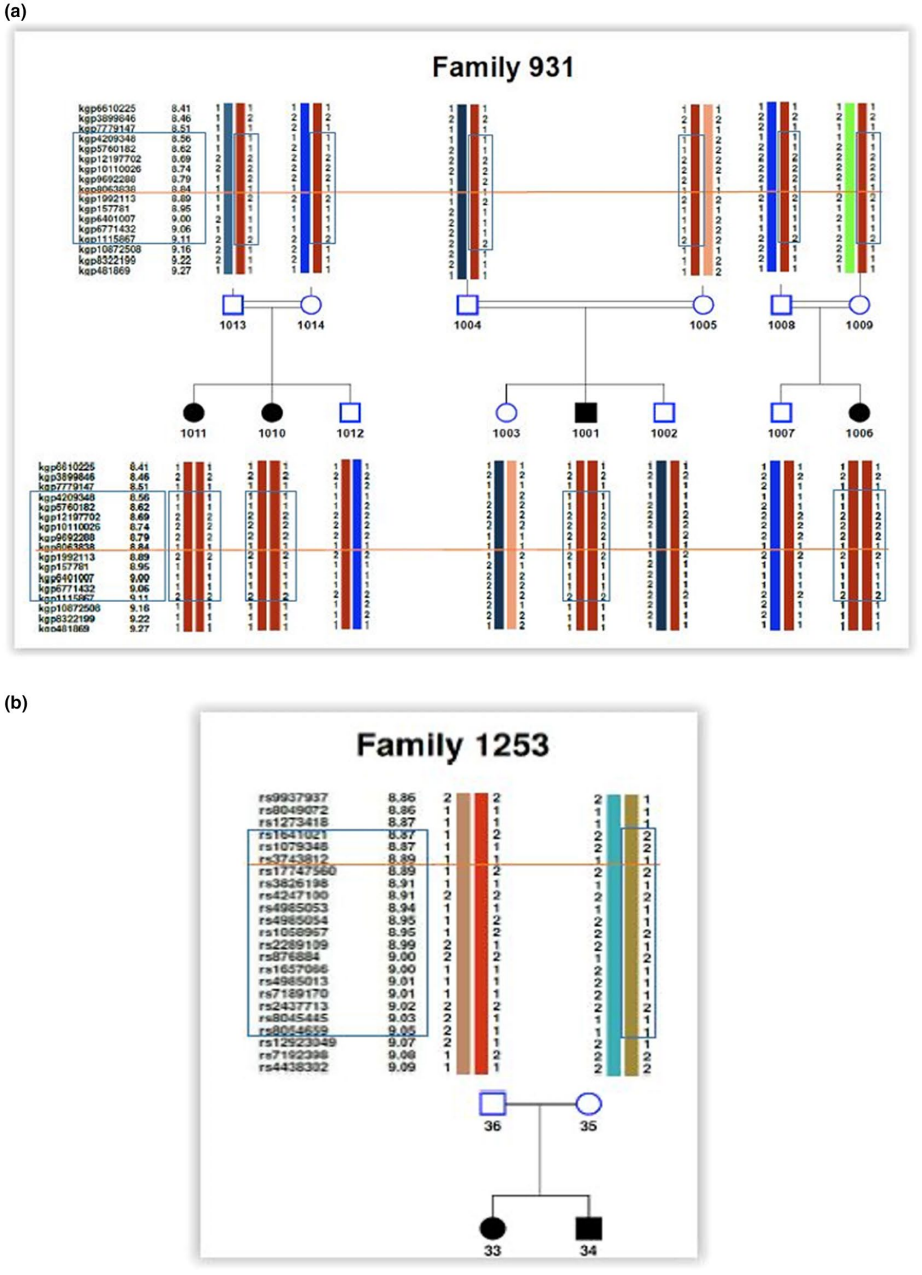
Haplotype reconstruction. Shown is the haplotype reconstruction for the (a) Spanish (Family 931) and (b) British family (Family 1253). For simplicity, only 2 generations are shown for the Spanish family. The blue box shows the disease‐associated haplotype (including the flanking markers used in the analysis). The orange line shows the position of the HIPKD promoter variant in relation to the other markers. The *kgp* identifiers shown for the Spanish families have been converted into the physical distance, based on the corresponding rs identifiers

We were able to identify a common haplotype, segregating with the mutation, in all families, and overlapping between the Spanish and British families (Figure 1).

For the Spanish families, haplotype analysis revealed a common haplotype of 0.312 Mb in size, located between positions 8.69 and 9.00 Mb on chromosome 16. This haplotype segregated with the disease allele and was observed in homozygous state in the four patients in family 931 and in heterozygous state in all their parents. Additionally, it was observed in heterozygous state in the remaining two patients and in one each of their parents from families 932 and 933. The common haplotype was not observed in any of the control samples. Taking the flanking markers into consideration, the linked haplotype comprised nine genotyped SNPs (Table 1).

**TABLE 1:** Markers used for haplotype analysis: Spanish (top) and British/US families (bottom)

Reference ID	Chromosome position	Reference allele (1)	Alternate allele (2)	Ancestral allele
rs72766414	8,690,404	G	**A**	**G**
rs72766499	8,740,900	**C**	**T**	**T**
rs1273373	8,791,426	**C**	**A**	**A**
rs3815507	8,841,547	**G**	**C**	**G**
c.‐167G>T	**8,891,573**			
rs8052077	8,893,836	**G**	**A**	**A**
rs1657067	8,947,077	**G**	**A**	**A**
rs4985065	9,002,672	**C**	**A**	**C**

Reference ID	Chromosome position	Reference allele (1)	Alternate allele (2)	Ancestral allele
rs3743812	8,886,271	**A**	**C**	**A**
c.‐167G>T	**8,891,573**			
rs17747560	8,892,154	**A**	**G**	**A**
rs3826198	8,906,539	**C**	**A**	**A**
rs4247100	8,912,593	**T**	**C**	**T**
rs4985053	8,943,551	**C**	**T**	**C**
rs4985054	8,945,568	**G**	**A**	**G**
rs1058967	8,947,036	**T**	**C**	**C**
rs2289109	8,992,828	**G**	**T**	**G**
rs876884	8,996,788	**C**	**T**	**C**
rs1657066	9,000,085	**A**	**C**	**A**
rs4985013	9,008,475	**G**	**A**	**A**
rs7189170	9,014,385	**G**	**A**	**G**
rs2437713	9,020,809	**T**	**G**	**T**

Bold indicates the variant examined.

In the British/US set, the common haplotype stretched over a region of 0.134 Mb (8.89–9.02 Mb on chromosome 16) and consisted of 13 SNPs. The haplotype was observed in heterozygous state in all the patients and their carrier parents. As before, the common haplotype was not observed in any of the control samples. Taking the flanking markers into consideration the linked haplotype comprised 15 genotyped SNPs (Table 1).

## 4 Estimating the age of the promoter variant

**FIGURE 2:**
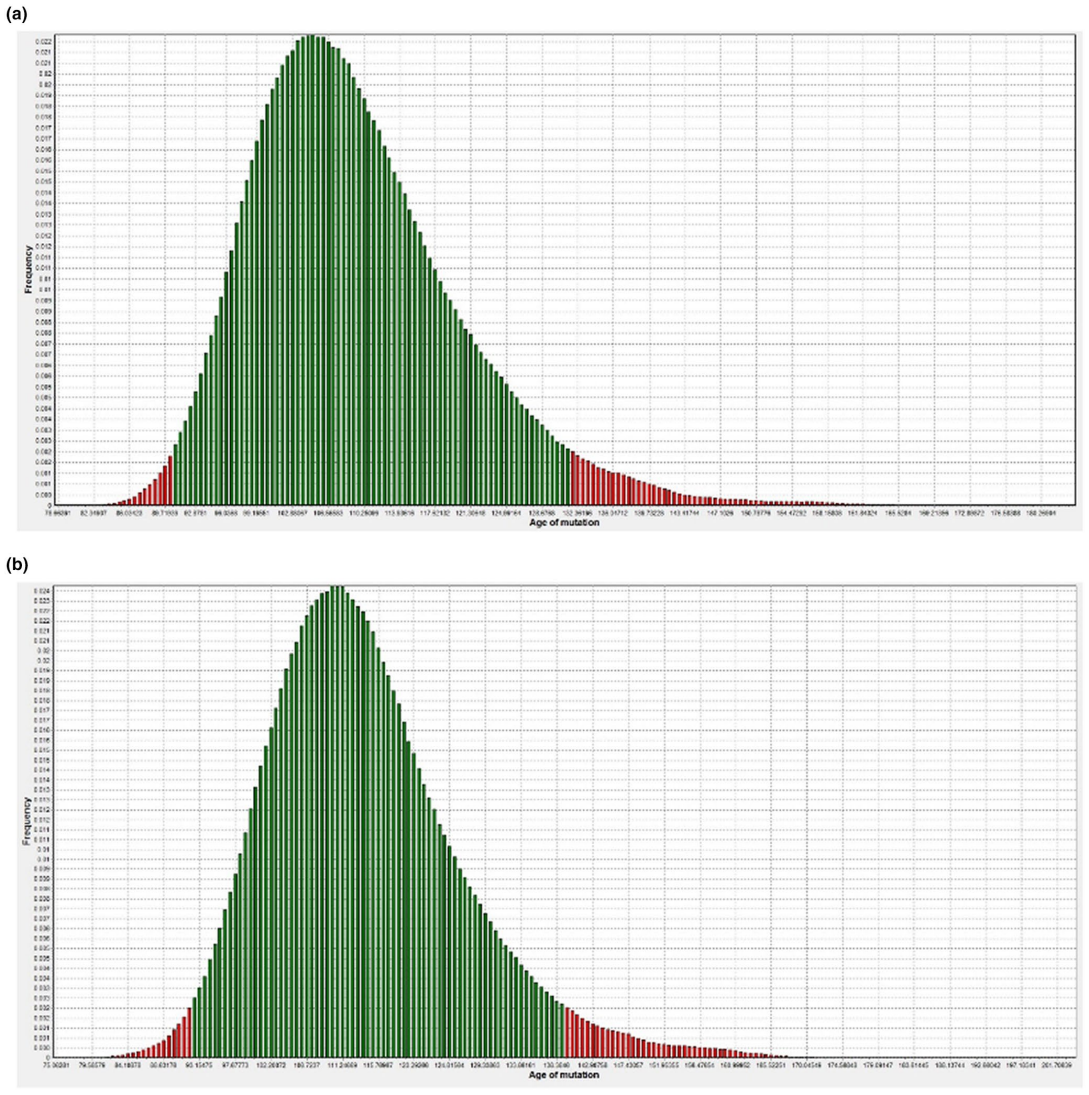
Age of founder mutation. The histogram produced by the *DMLE*+ software displaying the estimated age of the founder mutation in the *PMM2* promoter in the (a) Spanish and (b) a British family for an estimated incidence of HIPKD of 1 in 1,000,000. The estimated peak age is 105 generations (the green bars show the 95% confidence interval between 91 and 131 generations)

The age of the promoter variant in the Spanish families was estimated to have occurred 105 generations ago (rounded to the nearest generation) with a 95% confidence interval (CI) between 91 and 131 generations. Taking each generation as corresponding to 25 years, the mutation was estimated to have occurred 2625 years ago (95% CI 2275–3275 years). The results obtained from the DMLE+ software for the Spanish set are shown in Figure 2a.

Similarly, the age of the promoter variant in the British/US families was estimated to have occurred 110 generations ago (rounded to the nearest generation) with a 95% confidence interval between 93 and 139 generations ago.

This variant, taken all data together, is therefore estimated to have occurred 2750 years ago (95% CI 2325–3475 years). This is shown in Figure 2b.

## 5 DISCUSSION

The presence of a recently identified promoter variant in *PMM2* (c.‐167G>T) among European patients with HIPKD suggests that a founder effect may be responsible for the presence of this variant.

Haplotype analysis distinguished a common disease haplotype in the British/US as well as the Spanish patients’ sets. The specific arrangement of alleles observed in the patients was not seen in any of the European controls. The presence of a common haplotype that is overlapping in both the Spanish and the British/US families suggests that all the families have a common ancestor in whom the variant first arose.

This study provides evidence that the promoter variant is likely to have arisen around 600 BC. This corresponds to a period marked by intense migration within Europe. The Celtic population had expanded from central Europe and covered most of modern‐day Europe (Burton, 1979). Around approximately 500 BC, Germanic tribes had invaded many of these regions. Therefore, we speculate that the promoter variant in HIPKD arose in an ancestor who originated from central Europe.

All methods currently available to estimate the mutation age require assumptions to be made about the genetic and demographic parameters of the disease. First, we estimated the incidence of HIPKD to be around 1 in 1,000,000. Using this estimation, we would expect to observe 500 cases of HIPKD across Europe. Cabezas et al. reported only 17 patients with HIPKD which suggests that there are either many undiagnosed individuals yet or that the true incidence of HIPKD is even rarer than has been estimated in this study. The former may be possible since HIPKD is a newly reported disease and more individuals with HIPKD may be diagnosed as the disease becomes more recognised in the scientific and medical community. Indeed, subsequent families have been described and it is possible that some patients previously considered to have ARPKD but without genetic confirmation may now have their diagnosis corrected to HIPKD (Moreno Macián F et al., 2020; Soares et al., 2020). In fact, the phenotypic range associated with these promoter mutations may extend beyond HIPKD (Dorval et al., 2021). If, with time, the true incidence of HIPKD will become clearer, further studies could be carried out to refine the estimation of the age of the mutation.

Second, we estimated the population growth rate at 0.109. It is virtually impossible to verify the demographic parameters and as such, it is difficult to determine the mutation age accurately. Greenwood et al. have previously discussed the variability of results produced by DMLE+ when the growth rate is altered (Greenwood et al., 2010). While census data for the European population is available, the oldest available data is from 1960. Therefore, to obtain a better estimation of the population growth rate, alternative sources that give older approximations of the population size from the early 1900 s were used (Morris, 1918).

## 6 CONCLUSION

The promoter variant implicated in the development of HIPKD is associated with a common haplotype in all the affected individuals. Our findings support the hypothesis of a founder effect within the European population. The variant is likely to have arisen in a common founder in Europe over 2600 years ago. These results suggest that patients of European descent presenting with kidney cysts and hypoglycaemia due to hyperinsulinism may carry this promoter variant. Consequently, PMM2 and its promoter should be included in the genetic analysis of these patients to enable a specific diagnosis and appropriate genetic counselling. Since HIPKD is a newly recognized disease, future work may reveal other variants and patients of non‐European ethnicity.

## CONFLICT OF INTEREST

The authors declare no conflict of interest.

## AUTHOR CONTRIBUTIONS

RK, HS, DB and DI conceived the study, LGW, SF, SE, DB and KH contributed patient data, SI, MT and DI led data analysis and all authors participated in writing and revising the manuscript.

## Data Availability

Summary data is available in an anonymised format through collaboration with the authors.
